# Supplementary material for: A Global Screen for Assembly State Changes of the Mitotic Proteome by SEC-SWATH-MS
Source: Cell Syst. 2020 Feb 26;10(2):133–155.e6. doi: 10.1016/j.cels.2020.01.001 (PMC7042714; doi:10.1016/j.cels.2020.01.001)

P41229 | KDM5C_HUMAN | KDM5C DXS1272E JARID1C SMCX XE169
Monomer MW [kDa]: 175.72 Monomer expected elution fraction: 35

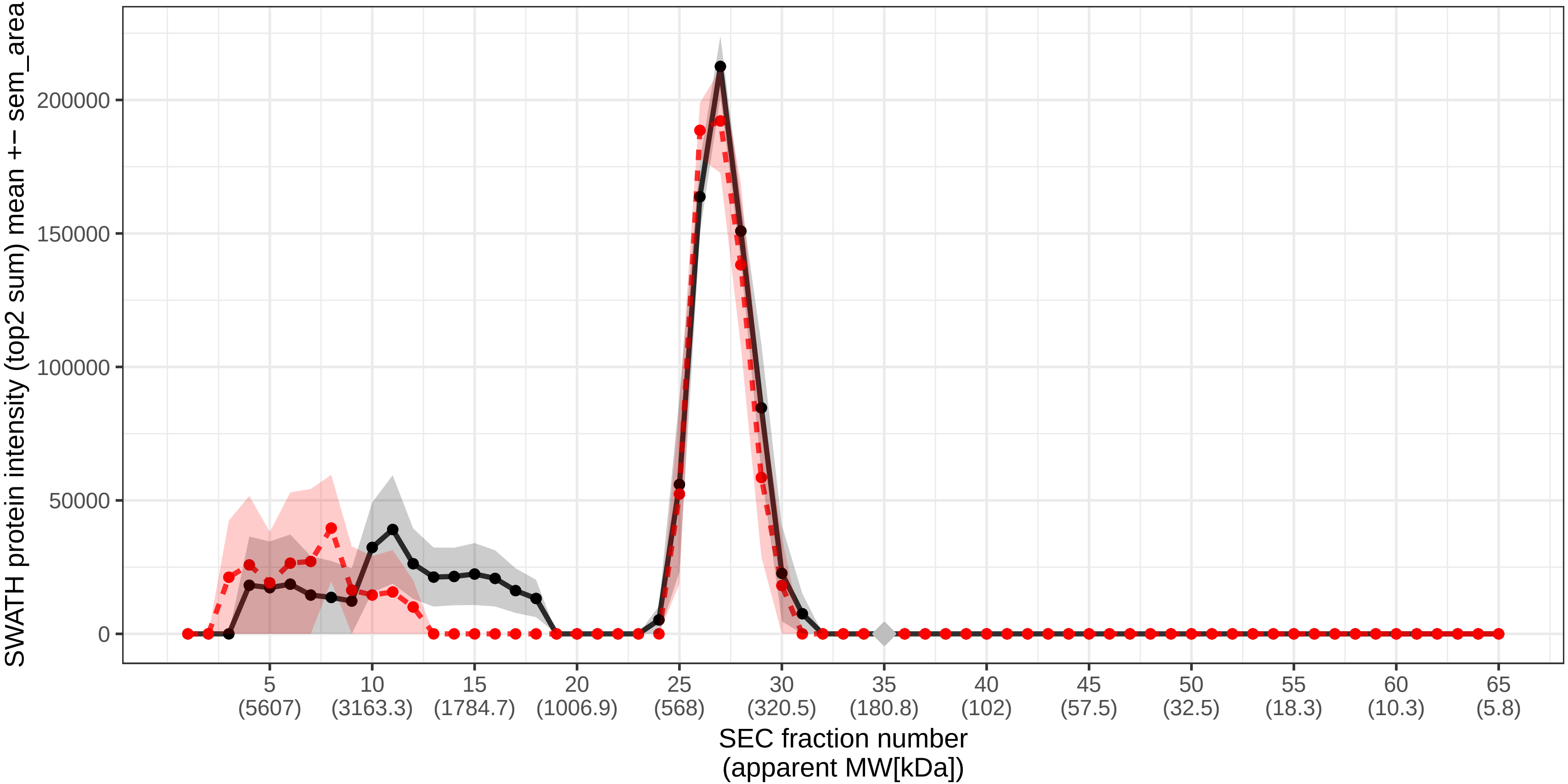

Supplement: Data S1. SEC-SWATH-MS Protein Chromatograms, Related to Figure 1 [file mmc6.zip › SECchrom_P41229_KDM5C_HUMAN_KDM5C_DXS1272E_JARID1C_SMCX_XE1.pdf]
